# Design of Acoustical Bessel-Like Beam Formation by a Pupil Masked Soret Zone Plate Lens

**DOI:** 10.3390/s19020378

**Published:** 2019-01-17

**Authors:** Daniel Tarrazó-Serrano, Sergio Castiñeira-Ibáñez, Oleg V. Minin, Pilar Candelas, Constanza Rubio, Igor V. Minin

**Affiliations:** 1 Centro de Tecnologías Físicas, Universitat Politècnica de València, Camí de Vera s/n, 46022 València, Spain; dtarrazo@fis.upv.es (D.T.-S.); sercasib@upvnet.upv.es (S.C.-I.); pcandelas@fis.upv.es (P.C.); 2 Radiophysical department, Tomsk State University, 30 Lenin Avenue, Tomsk 634050, Russia; oleg.minin@ngs.ru; 3 Engineering school of nondestructive testing, Tomsk Polytechnic University, 36 Lenin Avenue, Tomsk 634050, Russia

**Keywords:** ultrasonic lens, apodization, soret zone plate

## Abstract

The image performance of acoustic and ultrasound sensors depends on several fundamental parameters such as depth of focus or lateral resolution. There are currently two different types of acoustic diffractive lenses: those that form a diffraction-limited spot with a shallow depth of focus (zone plates) and lenses that form an extended focus (quasi-Bessel beams). In this paper, we investigate a pupil-masked Soret zone plate, which allows the tunability of a normalized angular spectrum. It is shown that the depth of focus and the lateral resolution can be modified, without changing the lens structure, by choosing the size of the pupil mask. This effect is based on the transformation of spherically-converging waves into quasi-conical waves, due to the apodization of the central part of the zone plate. The theoretical analysis is verified with both numerical simulations and experimental measurements. A Soret zone plate immersed in water with D/2F = 2.5 and F = 4.5λ changes its depth of focus from 2.84λ to 5.9λ and the lateral resolution increases from 0.81λ to 0.64λ at a frequency of 250 kHz, by modifying the pupil mask dimensions of the Soret zone plate.

## 1. Introduction

The necessity of manipulating waves has been one of the main objectives of the scientific community. Lenses are devices that achieve, through different physical phenomena such as refraction or diffraction, beam focusing and modulating effects. In the acoustic field, lenses have been developed for different areas such as biomedicine, engineering, and industry. One of the reasons why lenses are used in different fields is to reduce costs, since the use of lenses prevents the manufacture of new transducers [1,2,3,4,5,6,7,8,9,10,11]. Due to the interest that these lenses have gained, their design and improvement is currently a research subject. One type of acoustic lens changes the refractive index between media, known as gradient-index lenses, using labyrinthine structures [12,13,14]. Phononic crystals [15], acoustic metamaterials [16], and acoustic resonators [17] have also been used in the implementation of acoustic lenses. Further, another type of acoustic lens, which is proposed to be used in High Intensity Focused Ultrasound (HIFU) techniques, bases its design on a double foci to maintain the aperture of a transducer while reducing the f-number and the depth of focus. In this way, tissue coagulation can be induced in a smaller volume [18]. It is also important to consider the Side Lobe Level (SLL) parameter in ultrasound beamforming, because this parameter influences the image resolution. Regarding this parameter, a new type of lens called a super-oscillatory acoustic lens has been developed. This type of lens is proposed for image diagnosis due to the fact that it can focus at great distances and can minimize the SLL parameter, achieving an improved resolution [19].

A Fresnel Zone Plate (FZP), as a simple example of an acoustic lens, has been used in this study due to its dimension and its fabrication advantages [20,21,22]. FZPs are constructed alternating opaque and transparent acoustic rings and use the diffraction phenomenon to modulate and focus acoustic waves. These classic FZPs are also called Soret Zone Plates (SZPs) [23]. Calvo et al. developed and characterized an SZP for underwater ultrasounds, alternating opaque and transparent zones made of soft silicone rubber [24]. Results showed a main lobe widening, but also a small reduction on the first side lobes. Other studies show that acoustic field diffraction produces a maximum pressure field along the longitudinal axis. Nonetheless, secondary lobes can produce focal point resolution ambiguity [25,26,27].

In the ultrasound imaging technologies that require high resolution at the focus, the −3 dB main lobe width ($R_{lat}$) and the Depth of Focus (DoF) are the main factors that affect the quality of the image. In order to improve the lateral resolution ($R_{lat}$), it is necessary to increase the SLL, concentrating the energy at the focus and increasing the resolution. SZP lenses have a reduced DoF because they concentrate the energy in a much reduced zone along the longitudinal axis [28]. This can be a drawback in some applications, where a wider DoF is required. Moreover, SLL is rather poor in SZP lenses and could also be improved. For an arbitrary circular aperture at the focus, simplified expressions for lateral resolution, $R_{lat}$, and (DoF) are given by [29]:

(1) $$R_{lat}=\alpha \lambda \frac{F_{L}}{D}=\alpha \lambda f^{\#}$$

(2) $$DoF=\beta \lambda {\left(\frac{F_{L}}{D}\right)}^{2}=\beta \lambda f^{\#2}$$

where α is the light gathering angle, β is light diffraction angle, λ corresponds to the wavelength, $F_{L}$ is the focal length, *D* is the diameter of the lens, and $f^{\#}$ is the f -number, defined as the ratio of the focal length to the lens diameter. The β parameter is typically two in these lenses [29,30]. Equation (1) is also valid in the focal area for a focused lens with a high numerical aperture [31]. One could see that depending on the choice of frequency and $f^{\#}$, the lateral resolution can vary over several orders of magnitude. Combining Equations (1) and (2), a relation between $R_{lat}$ and DoF is obtained:

(3) $$DoF=\frac{\beta }{\alpha^{2}\lambda }R_{lat}^{2}$$

If conventional lenses are used, it is impossible to increase the DoF and the lateral resolution at the same time, as follows from Equation (3). For this reason, many papers investigated acoustic Bessel beams. The central core spot size of the Bessel beam is determined by the zeros of a Bessel function. If Bessel beam-based lenses are used, DoF and $R_{lat}$ become independent parameters, as shown in Equations (4) and (5).

(4) $$R_{lat}=\frac{2.4048}{k_{r}}=\lambda \frac{2.4048}{2\pi \beta }$$

(5) $$DoF=z_{max}=\frac{D}{2\tan\beta }$$

Thus, the $R_{lat}$ does not depend anymore on the lens diameter. On the other hand, DoF depends on the diameter of the lens and the diffraction angle [32].

A flat acoustic lens with an aperiodical structure that transforms a divergent beam into a Bessel-like beam has been reported in [33]. Bessel beams of sound waves have also been reported in [34,35]. However, they are not as broadly applied in acoustics as in optics, which is perhaps related to the lack of convenient formation techniques of such a kind of acoustic wave. Acoustic Bessel beams have been excited using acoustical axicons [36], in analogy to the optical case. In any case, the most convenient way to form acoustic Bessel beams is by using annular transducer arrays [37,38]. The formation of an acoustic Bessel-like beam by using an axisymmetric grating of rigid tori was reported in [39]. It could be mentioned that if Babinet’s principle is considered, both approaches (SZP and rigid tori scatterers) are considered equivalent.

This work is based on the study carried out by Minin et al. in the field of electromagnetism [40] (see Equation (7)). In that study, a modification of the distribution of the energy at the focus was proposed. The authors showed that by choosing a reference radius smaller than the first FZP radii, the SLL ratio was improved [41,42]. These results have been transferred to the ultrasonic field by Tarrazó-Serrano et al. [43]. Besides, the concept of the reference radius was independently used in acoustics in [44], but without analyzing the image quality. When a reference radius is larger than the first radii of the SZP equal to the equivalent design, this can be expressed as an SZP lens with a pupil mask, with the radius length equal to the reference radius ($r_{0}$).

The present work proposes and proves a new technique for acoustic quasi-Bessel beam formation using a planar structure based on SZPs [23] by adding a Pupil mask to the SZP (PSZP). This PSZP presents an elongated focus while improving the $R_{lat}$ simultaneously. Although SZPs in general and PSZP in particular are designed for a specific operating frequency, it has been demonstrated that SZP can also work at a range of different frequencies around the design frequency. In this case, there is a linear dependence between the focus location and the operation frequency [45]. Therefore, they could be used for imaging and therapeutic applications. It can be affirmed that, under specific conditions, part of a diffracted wave collimates, producing an elongated focus. Numerical calculations using the Finite Element Method (FEM) of acoustic waves propagating through such lenses were used to observe the complete acoustic field.

## 2. Mathematical Method and Simulations

SZPs are based on ring sections known as Fresnel regions. The purpose of the opaque rings is to block the destructive contributions to the focus. SZPs’ building parameters are the focal length ($F_{L}$), the number of Fresnel zones (*N*), and the wavelength of the signal (λ). The following equation provides the Fresnel lens radii for plane wave incidence [24]:

(6) $$r_{n}=\sqrt{n\lambda F_{L}+{\left(\frac{n\lambda }{2}\right)}^{2}}\;\;\;\;n=1,2,3,\ldots ,N$$

In order to design the size of the pupil, the reference radius ($r_{0}$) has been introduced in the construction equation of the Fresnel lens. This is arbitrarily defined, taking into account that, from the point of view of geometrical optics, the properties of the zone plate are not modified [40]. Nevertheless, this parameter is equivalent to modifying the reference phase in the wave approximation [42] and introduces the quasi-Fresnel radii concept. These radii are defined, for incident plane waves, by the following equation:

(7) $$r_{n}=\sqrt{n\lambda \sqrt{F_{L}^{2}+r_{0}^{2}}+{\left(\frac{n\lambda }{2}\right)}^{2}+r_{0}^{2}}\;\;\;\;n=1,2,3,\ldots ,N$$

The study of the physical phenomena involved in the interaction between the lenses and the wave front requires a mathematical model that considers the boundary conditions of the problem. In the present work, FEM has been implemented using the commercial software COMSOL Multiphysics [46] to calculate the acoustic pressure distribution. This method generates a numerical solution by discretizing the model and solving the Helmholtz partial differential equation:

(8) $$\nabla \cdot \left(-\frac{1}{\rho_{0}}\left(\nabla p\right)\right)=\frac{\omega^{2}p}{\rho_{0}c^{2}}$$

where $\rho_{0}$ is the host medium density, *c* is the ultrasound velocity, ω is the angular frequency, and *p* is the acoustic pressure. One of the problems with the use of FEM, is the extensive usage of memory resources, and axisymmetric models have been considered to optimize these limitations. The axisymmetric model takes into account the problem geometry, simplifying the model and reducing the computational time. Underwater transmission is considered with sound speed propagation (c=1500 m/s) and water density ($\rho_{0}=1000$ kg/m${}^{3}$). The wavelength of the signal (λ) is 6 mm, which corresponds to a design frequency (*f*) of 250 kHz. This λ has been selected to ease the mechanization of the lenses in the experimental setup. Lenses have been modeled as fully rigid considering the Neumann condition, which specifies that the sound velocity at the boundary is zero. The exterior contours of the model emulate the Sommerfeld condition. This boundary condition avoids internal reflections. Mesh geometry is fixed in triangles with a minimum element size of λ/14 and a maximum element size of λ/8 to prevent numerical dispersion.

**Figure 1:**
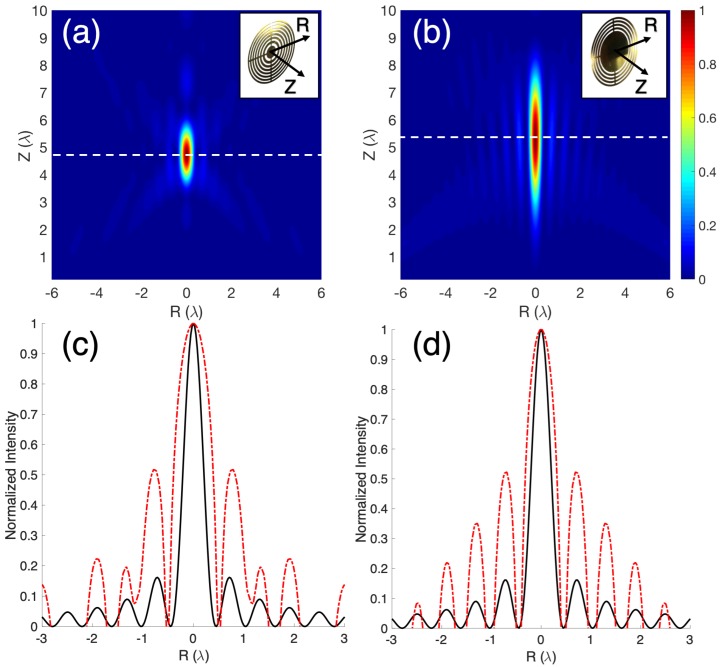
Self-normalized intensity maps to the maximum with the *R*- and the *Z*-axis normalized to λ for (**a**) a Soret Zone Plate (SZP) and (**b**) an SZP with amplitude Pupil mask (PSZP). Normalized intensity level focus transversal cuts from intensity maps (red dashed line) are compared to the Bessel $J_{0}^{2}$ function (black solid line) for (**c**) SZP and (**d**) PSZP.

In the current paper, lenses with high numerical aperture (NA) have been selected ($NA=1/2f^{\#}=D/2F_{L}=2.5$). The focusing profile of this device ($F_{L}=4.5\lambda$) presents a very compact beam with a short working distance. Figure 1 shows the normalized intensity maps and the axial focusing profiles for an SZP and a PSZP. Axes have been normalized with respect to the wavelength.

It can be observed from Figure 1a,b that the SZP and PSZP lenses present focusing profiles with different focal lengths and shapes. As expected, the PSZP has a more extensive focus area (larger DoF) than the SZP. Moreover, the PSZP has a characteristic structure similar to quasi-Bessel beams with intensity profiles that closely resemble the ideal $J_{0}^{2}$ transverse-intensity distribution of Bessel beams (Figure 1d), while SZP does not show this behavior.

**Figure 2:**
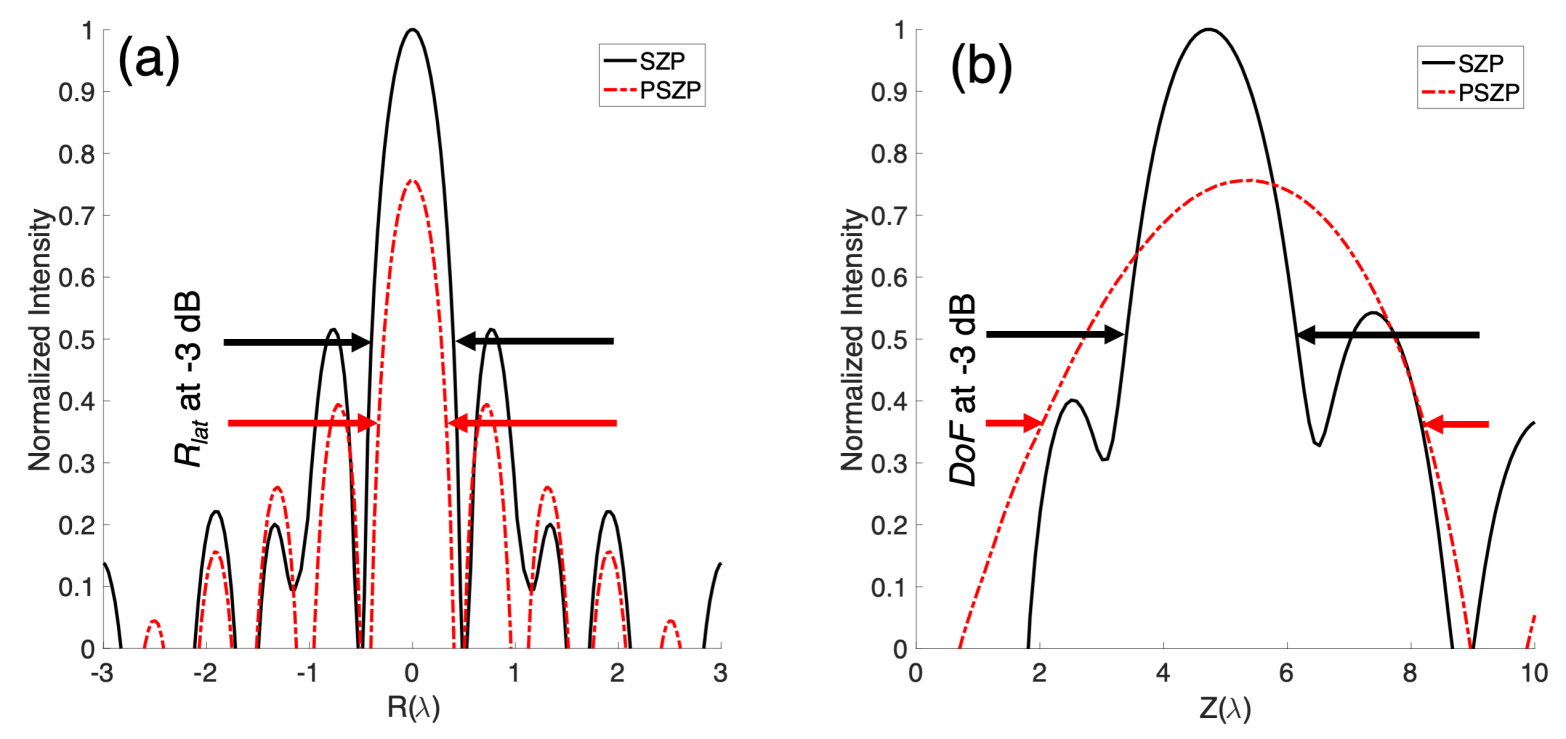
Numerical normalized intensity results for (**a**) the *R*-axis cut and (**b**) the *Z*-axis cut. The black solid line corresponds to classical Soret Zone Plate (SZP). The dashed-dotted red line corresponds to the amplitude pupil masked Soret Zone Plate (PSZP). Black and red arrows mark the DoF and $R_{lat}$ limits for SZP and PSZP, respectively.

The normalized intensity distributions along the longitudinal axis (*Z*-axis) and the radial axis (*R*-axis) are presented in Figure 2. It can be observed that a maximum normalized intensity value is obtained in the SZP case, the PSZP maximum value being equal to 0.76.

Table 1 shows DoF at −3 dB and $R_{lat}$ at −3 dB for both SZP and PSZP lenses. As can been observed from Table 1, DoF in PSZP is larger than in a classical SZP, while the resolution for point objects in the PSZP is better since the central maximum is narrower and it has a little more energy in the outer rings of the diffraction pattern compared to the central maximum. Therefore, the PSZP generates quasi-Bessel beams increasing the DoF and reducing the diameter of the central spot when the pupil mask becomes larger. It is worth mentioning that a paraxial study of this phenomenon in the optical field was performed in [29]. In the PSZP case, when the pupil becomes larger and more Fresnel zones are covered, the focus sharpens further in the radial direction, and the relative side lobe intensity is increased. Thus, the central zones play an important role in reducing the SLL, while the outer zones cause the central peak to sharpen. The diameter of the central maximum at the focal spot is less than the equivalent Airy disk. The DoF at −3 dB is the Full Length Half Maximum (FLHM) in the longitudinal axis, while $R_{lat}$ at −3 dB is equivalent to the FLHM along the transverse axis.

**Figure 3:**
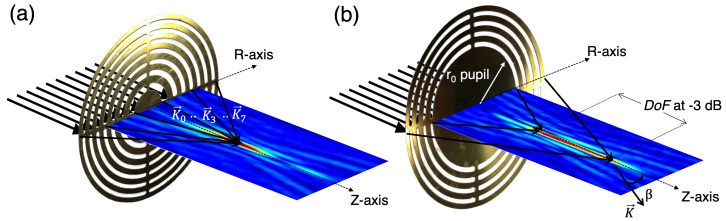
Beam formation scheme of (**a**) SZP and (**b**) PSZP.

The effects of extended DoF with a quasi-Bessel structure for PSZP may be described (to simplify the problem) as presented in Figure 3. The SZP lens consists of concentric dielectric rings, which can be treated as quasi-periodic gratings with different local grating constants at different radii. For single-point focusing, the normal incident wave is diffracted towards the designed focal point by these local gratings. The diffraction angles corresponds to the different radii and ensure the formation of a focal spot (Figure 3a). The normalized radial spatial frequency $k_{r}/k$ is related to the angle β as $k_{r}/k=\sin\beta$, where $k_{r}$ is the radial component of the wave vector *k* and β is the angle between the wave vector and the longitudinal axis. For an ideal Bessel beam, the values of $k_{r}$ and β are the same for all contributions. The range of the diffraction angle corresponds to the normalized angular spectrum bandwidth.

A PSZP can also be described as diffraction gratings because the Fresnel zones’ widths are almost identical. Thus, the local gratings diffract the incident waves towards different points on the longitudinal axis for a small range of diffraction angles (Figure 3b). The interference pattern is a 2D Bessel function of the first kind, located at the focal distance. Therefore, the local grating constant d(r) at radius *r* can be obtained using the grating equation, d(r)sinβ(r)=λ, where λ is the incident wavelength. Thus, the original SZP angular spectrum is controlled by the pupil mask size. It should be noted that the acoustic lens is immersed in water, producing a compressed angular spectrum according to the refractive index of a medium.

The focused wave is diffracted from the outer rings, and the first-order diffraction beams form the acoustical needle, which is similar to the formation of a quasi-non-diffracting beam with conical lenses. It should be noted that in the optical case, the pupil mask method was used to block the light in the point-focusing super-oscillatory lens to achieve a DoF as high as 5λ to 20λ. However, the achieving of such an extended DoF was at the expense of a degradation in the focus lateral resolution [47,48].

## 3. Experimental Results

**Figure 4:**
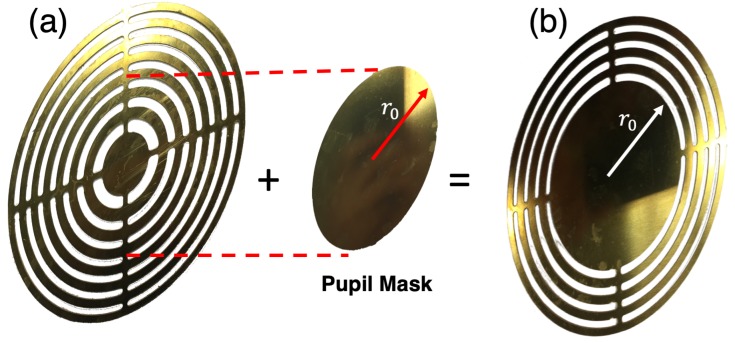
Implemented lenses in brass for the experimental results; where (**a**) corresponds to the experimental classical Soret Zone Plate (SZP) and (**b**) corresponds to the implemented Pupil masked Soret Zone Plate (PSZP).

The purpose of this work is to prove that an SZP with a Pupil mask (PSZP) is able to increase the DoF, reducing at the same time the $R_{lat}$. To carry out the implementation of both lenses, Equation (6) and Equation (7) have been considered for SZP and PSZP, respectively. The material selected for their construction was brass due to its low transmission coefficient. A reduced transmission factor allowed the lens rings to behave as a material opaque to sound. The SZP was mechanized applying construction conditions previously mentioned (Figure 4a). To construct the PSZP, Equation (7), which introduces the reference radius ($r_{0}$), was applied. The selected $r_{0}$ corresponds to the pupil radius, which blocked three Fresnel zones. As shown in Figure 4b, to ease the mechanization procedure, the PSZP was not built in two different parts (lens and pupil mask). Instead, the PSZP was mechanized as a single piece including the radius pupil $r_{0}$, which blocked three Fresnel zones.

All measures were done using an automated full-precision measurement system in order to validate and compare both lenses. This system consisted of a fixed ultrasonic transducer and a 3D positioned hydrophone [49], which granted precise and reliable results with 1 × 1 mm${}^{2}$ scanning. The transducer used in this experiment was a 250-kHz Imasonic piston with 32 mm of active diameter, and the needle hydrophone was an MPM1/1 from Precision Acoustic Ltd. made of polyvinylidene fluoride with a diameter of 1.5 mm. A flat transfer function between 0.2 and 15 MHz provided the accuracy of the measurements.

**Figure 5:**
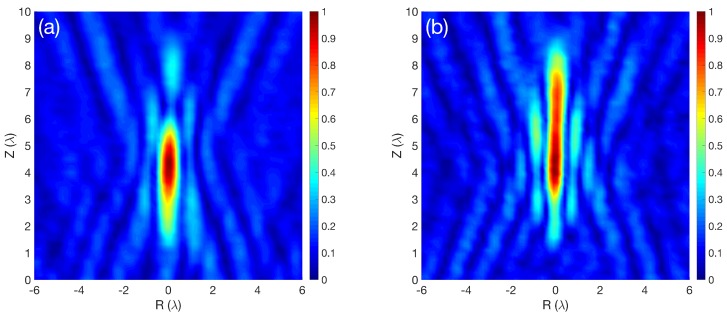
Experimental self-normalized intensity map results for the implemented lenses; where (**a**) corresponds to the experimental classical Soret Zone Plate (SZP) and (**b**) corresponds to the implemented Pupil masked Soret Zone Plate (PSZP).

Figure 5 shows the experimental self-normalized intensity maps for the SZP lens (Figure 5a) and the PSZP lens (Figure 5b). It can be seen that the pupil mask effect elongated the focus due to the diffraction grating lens behavior. In the SZP lens, as shown in Figure 5a, the focus was located at the designed $F_{L}$. In the PSZP case, the maximum energy was located at the same $F_{L}$ as in the SZP case, but the energy was distributed along the longitudinal axis with a needle shape. The PSZP focused beam was also narrowed with respect to the SZP case.

**Figure 6:**
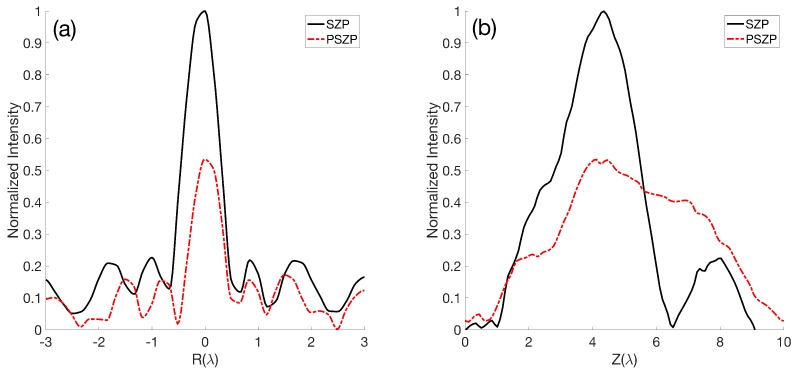
Experimental normalized intensity results for both implemented lenses. The axial and longitudinal axis are shown, where (**a**) corresponds to the *R*-axis cuts and (**b**) corresponds to the *Z*-axis cuts at the $F_{L}$ position

The longitudinal and axial cuts for both lenses were obtained from intensity maps. The intensity cuts were normalized with respect to the maximum value and are shown in Figure 6 in order to highlight the differences in levels and energy distribution in both lenses. Figure 6a corresponds to the radial or *R*-axis cut. It can be observed that the decreasing of the secondary lobes was more similar to a Bessel function in the PSZP case than in the SZP case. Figure 6b corresponds to the longitudinal cuts of both lenses along the *Z*-axis. Here, it can be observed that the pupil mask generated a longitudinal energy distribution as opposed to the SZP behavior.

Table 2 shows the experimental data obtained for the DoF at −3 dB and $R_{lat}$ at −3 dB for both lenses. When DoF values were compared, an increase of 2.21λ was observed. Therefore, an expansion of the focus in the longitudinal axis was achieved. In the case of the $R_{lat}$, the PSZP achieved a narrower beam. The use of the pupil mask increased the resolution by 0.12λ. By comparing experimental and numerical values (see Table 1 and Table 2), it can be observed that there was a good agreement between them. The discrepancies found between Figure 2 and Figure 6 could be explained by the fact that the ultrasound transducer did not emit plane waves. In such a way, the wavefront arriving at the lens was not a completely plane wave.

## 4. Conclusions

In this work, we have proposed an improvement over a classical SZP introducing a pupil mask. The design of a quasi-non-diffracting beam with a sub-wavelength transverse size was achieved using a PSZP. This design has been verified using numerical models and experimental measurements in controlled conditions. When an amplitude pupil mask was used, a quasi-Bessel beam was obtained instead of a diffraction spot focus. This effect was produced by modifying the spherically-converging waves into quasi-conical waves. Therefore, a quasi-Bessel beam distribution was obtained when a PSZP was used.

With the PSZP proposed in this paper, a spatial resolution enhancement from 0.81λ to 0.64λ was accomplished. In addition, DoF increased from 2.84λ to 5.94λ compared with the classical SZP. The experimental results were in good agreement with the numerical simulations. The obtained results allowed the modulation of the acoustic beam without modifying the lens. Therefore, the same SZP could be used for different targets by changing the pupil mask, and these results confer great versatility to SZP lenses. This type of PSZP has applications in different areas where a compromise between DoF and lateral resolution is required. As an example, in 3D imaging, ultrasonic sensors with a long depth of focus eliminate the need for depth scanning, making this technique considerably faster.

## Figures and Tables

**Table 1:** DoF at −3 dB and $R_{lat}$ at −3 dB, comparison of the numerical results.

	DoF at −3 dB (λ)	$R_{\mathrm{lat}}$ at −3 dB (λ)
SZP	2.84	0.81
PSZP	5.94	0.64
Bessel $J_{0}^{2}$	-	0.64

**Table 2:** DoF at −3 dB and $R_{lat}$ at −3 dB, comparison of the experimental results.

	DoF at −3 dB (λ)	$R_{\mathrm{lat}}$ at −3 dB (λ)
Experimental SZP	3.51	0.79
Experimental PSZP	5.72	0.67
